# Oncolytic Virotherapy for Hematological Malignancies

**DOI:** 10.1155/2012/186512

**Published:** 2011-10-29

**Authors:** Swarna Bais, Eric Bartee, Masmudur M. Rahman, Grant McFadden, Christopher R. Cogle

**Affiliations:** ^1^Division of Hematology and Oncology, Department of Medicine, College of Medicine, University of Florida, ARB R4-202, P.O. Box 100278, Gainesville, FL 32610-0278, USA; ^2^Department of Molecular Genetics and Microbiology, College of Medicine, University of Florida, ARB R4-295, P.O. Box 100266, Gainesville, FL 32610, USA

## Abstract

Hematological malignancies such as leukemias, lymphomas, multiple myeloma (MM), and the myelodysplastic syndromes (MDSs) primarily affect adults and are difficult to treat. For high-risk disease, hematopoietic stem cell transplant (HCT) can be used. However, in the setting of autologous HCT, relapse due to contamination of the autograft with cancer cells remains a major challenge. *Ex vivo* manipulations of the autograft to purge cancer cells using chemotherapies and toxins have been attempted. Because these past strategies lack specificity for malignant cells and often impair the normal hematopoietic stem and progenitor cells, prior efforts to *ex vivo* purge autografts have resulted in prolonged cytopenias and graft failure. The ideal *ex vivo* purging agent would selectively target the contaminating cancer cells while spare normal stem and progenitor cells and would be applied quickly without toxicities to the recipient. One agent which meets these criteria is oncolytic viruses. This paper details experimental progress with reovirus, myxoma virus, measles virus, vesicular stomatitis virus, coxsackievirus, and vaccinia virus as well as requirements for translation of these results to the clinic.

## 1. Hematological Malignancies 

Hematological malignancies include leukemias, lymphomas, multiple myeloma (MM), and the myelodysplastic syndromes (MDSs) that most often affect individuals older than 60 years of age. These blood cancers affect approximately 10% of Americans diagnosed with cancer each year, and an estimated 140,000 were newly diagnosed in 2010 (National Cancer Institute, Surveillance Epidemiology, and End Results). Unfortunately, despite best available therapies, an estimated 50,000 individuals died from these diseases in 2010. 

The causes of hematological cancers vary depending on the specific malignancy. Exposure to environmental toxins such as benzenes, prior cytotoxic treatment such as radiotherapy or chemotherapy for an antecedent cancer, as well as infections have all been implicated as causative factors in initiating hematological malignancies. In contrast, recurrent cytogenetic abnormalities have also been observed in hematological malignancies. These abnormalities often form the basis for assigning prognosis. For example, in acute myeloid leukemia (AML), recurrent mutations that portend for a high risk of relapse after conventional treatment include those with chromosome 7 abnormalities, chromosome 5 abnormalities, complex karyotypic abnormalities, and mutations in the *FLT3* gene. Genetic information can also indicate the most appropriate therapy. For instance, in patients with acute promyelocytic leukemia with the abnormal *PML-RARA* gene fusion, treatment with all transretinoic acid (ATRA) and cytotoxic chemotherapy can cure approximately 90% of patients [1]. In patients with MDS and deletion of chromosome 5q, treatment with lenalidomide can improve blood counts in 75% of patients [2]. 

Based on the utility of genetic information in determining prognosis and type of treatment in hematological malignancies, increased attention has been given to fully assessing the blood cancer genome. Recently, whole genome sequencing of an AML patient's DNA revealed several novel mutations never before associated with oncogenesis [3]. This technology also recently led to the discovery of *TET2* mutations as common gene mutations in MDS and emphasized the importance of epigenetic dysregulation in this disease [4, 5]. Because of the abnormal DNA methylation that occurs after *TET2* mutations, finding this mutation in an MDS patient's genome may indicate treatment with a hypomethylating agent such as azacitidine or decitabine [6]. Recently, whole genome sequencing was reported useful in determining the best treatment for a patient with AML [7]. Thus, genome analysis has the strong potential for personalized medicine in hematological malignancies. 

In some hematological malignancies, such as MDS, abnormalities in bone marrow stromal cells are believed to affect hematopoietic stem and progenitor cells, leading to neoplastic transformation [8]. Evidence that the bone marrow microenvironment is an important factor in the oncogenesis of hematological malignancies has spurred great interest in regulating microenvironmental interactions as a means for improved therapies. We have targeted blood vessels in the leukemia niche with the novel vascular disrupting combretastatin, OXi4503, and have successfully regressed disease [9]. This work has been translated into a phase I clinical study (http://www.ClinicalTrials.gov Identifier NCT01085656).

Cancer stem cells have been identified for some hematological malignancies [10]. In the specific case of acute myeloid leukemia (AML), a small subpopulation of cancer stem cells have been identified in the CD34+CD38−CD123+ fraction [11, 12]. In MM, myeloma stem cells have been found in the CD138− B cell fraction, which replicate and differentiate into CD138+ malignant plasma cells [13]. In chronic myeloid leukemia (CML), hematopoietic progenitor cells are believed to be the cancer-initiating cells which are endowed with cancer stem cell properties after acquiring the abnormal *BCR/ABL* gene fusion [14] 

## 2. Treatment of Hematological Malignancies

The cornerstone of conventional therapy for hematological malignancies includes agents that block cell division such as antimetabolites (e.g., cytarabine), DNA alkylating agents (e.g., cyclophosphamide), and anthracyclines (e.g., daunorubicin). Treatment with these agents induces initial remission in a high percentage of patients; however, relapsed disease remains a major challenge in treating patients with hematological malignancies.

For example, in cases of AML, remission rates with standard induction chemotherapy such as seven days of continuously infused cytarabine and three days of anthracycline bring about initial complete remissions in approximately 30–70% of patients. However, in older individuals, who are more commonly diagnosed with AML, long-term prognosis can be grim with only 10–20% of patients surviving without disease [15]. For patients with high risk AML, allogeneic HCT is used and can be curative in approximately 40% of patients. With this procedure, the donor immune system recognizes any residual leukemia in the recipient as foreign because of minor human leukocyte antigen mismatches and/or unique AML antigens, resulting in elimination and persistent surveillance for the malignant cells. By similar mechanisms, the donor immune system can recognize the recipient's normal organs (skin, gastrointestinal tract, liver, lungs, joints) as foreign and elicit graft versus host disease (GVHD). Although potentially curative, most AML patients due to their age-related comorbidities are not fit for the high risks associated with allogeneic HCT (e.g., GVHD, life-threatening infections, organ toxicity) and/or do not have a suitable allogeneic stem cell donor. Experimental therapies for AML have recently included specific mutation-targeting agents such as FLT3 inhibitors for patients with internal tandem duplications in the *FLT3* gene of the AML clone. However, results from these clinical trials have been disappointing. 

For patients with MM, treatment decisions are often based on risk for refractory and relapsed disease. Certain chromosome abnormalities, such as deletion of chromosome 13, portend for poor prognosis. In addition, gene expression profiling can be used to risk-stratify MM disease [16]. For patients with standard risk MM, initial treatment is dependent on the patient's eligibility for high-dose chemotherapy followed by autologous HCT, which can prolong disease-free and overall survival but carries treatment-related risks of organ toxicity, need for transfusions, and life-threatening infection. Patients eligible for autologous HCT are treated with nonalkylating agent induction therapies such as thalidomide and dexamethasone or lenalidomide and dexamethasone [17, 18]. After this initial therapy, patients have the option of early versus delayed autologous HCT. If early HCT is used, then a second HCT can be performed in patients who do not achieve a very good partial remission or better [19]. If the patient elects for delayed high-dose chemotherapy followed by autologous HCT, then transplant is not performed until initial induction therapy brings about a plateau in response or progressed disease develops. Even using these treatments, however, autologous HCT rarely brings about cures for MM, as the disease nearly always relapses. 

## 3. Autograft Contamination and Disease Relapse after Transplant

Despite the significant increase in use of autologous HCT for hematologic malignancies, disease relapse is a primary cause of death after transplant. Graft contamination is thought to be the chief reason for posttransplant relapse. This premise is supported by multiple lines of evidence. First, transplant of HSPC from syngeneic (identical twin) donors leads to lower incidences of disease relapse in patients with multiple myeloma, low-grade non-Hodgkin's lymphoma, AML, and ALL [20–22]. Second, numerous reports show that transplanted autografts contain minimal residual disease (MRD) in a variety of patients with cancer [23–39]. The level of MRD, detected by flow cytometry, immunohistochemistry, and molecular methods, directly correlated with risk of disease relapse and death. Whereas these lines of evidence show a strong correlation, direct proof of contaminated autografts through tracing studies are most compelling. Thus, the third line of evidence comes from gene marking studies [40–42]. In these clinical studies, autologous HSPCs were genetically tagged and then transplanted. Relapsed disease was evaluated for the tag. In a variety of leukemias and cancers, the posttransplant relapsed disease contained the pretransplant tag. Together, these lines of evidence support the premise that contaminating cells within the autologous transplant graft can be the origin of relapsed disease after transplant.

## 4. Purging Strategies

Considering the high rates of refractory and relapsed in patients with hematological malignancies and evidence of contaminating cancer cells in autologous HCT grafts, it is possible that graft purging of contaminating cells may improve posttransplant disease-free and overall survival. Ideally, a safe and effective purging strategy should specifically target the contaminating malignant clone and spare normal HSPC needed for reconstitution of immunity, erythropoieis, and platelets.

Several purging strategies have been attempted to selectively target malignant cells from autologous HCT grafts. One strategy is to treat the autologous graft after collection but prior to transplant back into the patient. A number of these *ex vivo* purging techniques have been tested such as:

- chemotherapy with antiproliferative drugs such mafosfamide and 4-hydroperoxycyclophosphamide (4-HC, active metabolite of cyclophosphamide) [43, 44];
- CD34+ stem/progenitor cell enrichment using immunomagnetic selection [45];
- immunotoxins or hybrid cytotoxic proteins designed to selectively kill cancer cells such as heregulin (HRG) *Pseudomonas* exotoxin (PE) 40 [46];
- immunomagnetic removal of tumor cells when the tumor cells express a unique antigen [47];
- monoclonal antibodies such as alemtuzumab (anti-CD52) and rituximab (anti-CD20) [48];
- photodynamic purging by rhodamine [49].

Unfortunately most of these *ex vivo* purging techniques also impaired normal hematopoietic stem and progenitor (HSPC) function and therefore have not translated to routine clinical practice for autologous HCT [50]. Purging strategies utilizing cytotoxic chemotherapy can be nonselective to cancer cells, and HSPC can be susceptible to the cytotoxic drugs [43, 44]. Immunomagnetic selection based on one cell surface marker (i.e., CD34) can enrich for normal HSPC; however, this selection process is never 100%, and the positive fraction may contain contaminating cancer cells [45, 47]. Moreover, discriminating normal from malignant HSPC can be difficult when using just cell surface markers because the two populations are sometimes indistinguishable by immunophenotyping. Given similarities in immunophenotype, immunotoxins may target both malignant and normal HSPC, leading to impaired normal hematopoiesis and posttransplant hematopoietic reconstitution [46, 48].

Ideally, *ex vivo* purging is selective for the cancer cells yet spares normal HSPC. Moreover, the ideal purging technique should be applied quickly (within minutes-to-hours) so that the transplant process is not delayed and any modifications to standard transplantation protocols are minimized. Cell viability is a time-dependent variable, and the quicker the manipulation the higher cell viability for transplant. In the postthaw setting, cell viability can diminish within hours, thus purging techniques applied to thawed products should be especially time sensitive in order to provide patients with the highest cell viability for transplant.

## 5. Oncolytic Virotherapy for Hematological Malignancies

**Figure 1:**
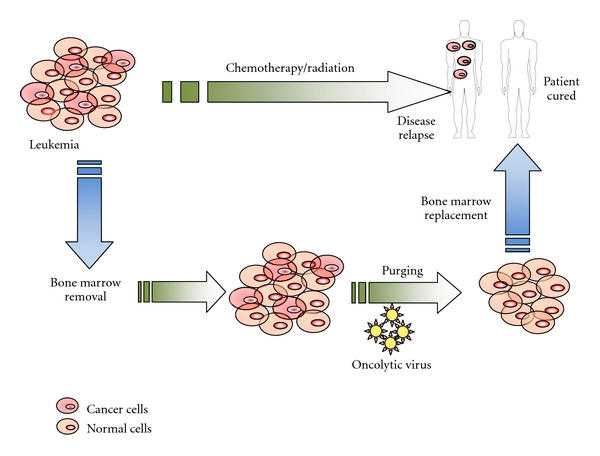
Proposed treatment schema of oncolytic virotherapy for patients with hematological malignancies undergoing high-dose chemotherapy and autologous HCT.

Oncolytic viruses may meet criteria as ideal purging agents for hematological malignancies. Specifically, certain oncolytic viruses selectively target malignant hematopoietic cells such as multiple myeloma and leukemia cells while sparing normal HSPCs [51]. This capacity to purge autologous HCT grafts makes oncolytic viruses particular attractive for potential use in the clinical transplant setting (Figure 1). A few oncolytic viruses have already been translated into the clinic (Table 1).

One potential purging agent is coxsackievirus A21 (CVA21) based on its ability to selectively target hematological malignant cells [52]. CVA21, a common enterovirus, exhibited a potent cytostatic and cytocidal effect against three MM cell lines with reduced cytotoxicity against normal human peripheral blood mononuclear cells (PBMCs) [53]. CVA21 specificity is believed to be related to expression of intercellular adhesion molecule-1 (ICAM-1) and decay accelerating factor (DAF) on the surface of target cells. While the immunocompromised status of MM patients receiving chemotherapy poses a concern for the use of virotherapy, it may be in these patients that CVA21 virotherapy will have the most successful outcome due to the lack of antiviral immunity. Disseminated CVA21 infection can be controlled by antiviral compounds, such as pleconaril [54] or immunoglobulin [55]. CVA21 has already been administered to end-stage melanoma patients without adverse effects [56], and further human trials are currently underway to evaluate safety. 

Another potential oncolytic virus for the treatment of hematological malignancies is reovirus [57]. Reovirus is a double-stranded RNA virus that is replication competent and preferentially infects cells with hyperactivated signaling, for example, in the Ras pathway. When reovirus was used to *ex vivo* purge MM cells from admixtures of apheresis products, purging was incomplete: only 50% of the MM cells were effectively purged. Also, reovirus was unable to purge follicular lymphoma and Burkitt's lymphoma cells [58]. A major advantage with reovirus is that it does not affect normal HSPCs. Therefore, reovirus may have potential in certain hematological malignancies, but it remains to be defined how clinically effective the virus is at eliminating each type of cancer.

Vesicular stomatitis virus (VSV) is another virus with oncolytic potential [59]. This negative strand RNA virus lacks toxicity for HSPCs in culture and has oncolytic activity against AML cell lines. Moreover, VSV can purge MM from mobilized PBSC CD34+ cells [60]. 

The Edmonston-B vaccine strain of measles virus (MV-Edm) also has reported oncolytic activity against MM. Using six clinical MM samples and a transplant model into immunodeficient mice, this measles virus successfully purged myeloma cells [61]. The intrinsic tumor selective cytotoxicity is an attractive feature of this agent. They also noted that administration of MV-Edm into MV-susceptible transgenic mice expressing the human CD46 receptor resulted in infection of macrophages in spleen, lymph nodes, and peritoneal cavity [62]. To enhance virus specificity, they generated an anti-CD38 scFv and demonstrated that display of scFv redirected virus binding and entry into CD38 receptor positive cells that were devoid of natural measles receptors [63]. The MV-Edm virus is currently in a phase I clinical study for recurrent or refractory MM where it is administered systemically via intravenous route along with cyclophosphamide chemotherapy (http://www.ClinicalTrials.gov ID NCT00450814) [64]. In this trial, the investigators are using the MV-NIS Edmonston lineage which was genetically engineered to express the human sodium iodide symporter (NIS). Insertion of the NIS protein into MV enables pharmacokinetic monitoring of the virus by means of radioactive iodine (^123^I) administration. Cells infected with MV-NIS will show increased uptake of the radioactive iodine, and this uptake can be serially tracked in real time. The patient's normal thyroid function is protected by coadministration of a normal thyroid hormone, triiodothyronine (T3). 

Live attenuated measles virus (MV) has potent oncolytic activity against MM tumor xenografts. The virus is tumor selective and preferentially targets cells that express high levels of CD46 receptors [65]. A vaccine strain of MV causes regression of large established human lymphoma xenografts in immunodeficient mice. MV is a negative-strand RNA virus, and, interestingly, the presence of anti-MV antibodies does not compromise the oncolytic effect of MV [66].

Adachi et al. reported a midkine promoter based conditionally replicative adenovirus (Ad) for the treatment of pediatric solid tumors and bone marrow tumor purging. A conditionally replicative Ad in which the expression of E1 is controlled by the MK promoter achieved high levels of viral replication in neuroblastoma or Ewing's sarcoma cells and induced tumor cell killing. No damage to CD34+ cells was seen, even after three hours of infection at 1000 MOI [67]. Adenovirus serotype 5 (Ad5) and other low-seroprevalence adenoviruses may have utility as oncolytic agents against MM and other hematological malignancies [68]. 

Tumor-specific double-deleted Vaccinia virus has also been tested in multiple myeloma [69]. Esfandyari et al. were the first to document permissiveness of lymphoma cells to oncolytic herpes viruses and introduced ELK as a suitable factor for predicting tumor susceptibility to novel anticancer agents [70]. 

Oncolytic rat parvovirus, H-1PV, may be a potential candidate for the treatment of some non-Hodgkin's B-cell lymphomas, including those resistant to apoptosis induction by rituximab. H-1PV efficiently killed through necrosis while sparing normal B lymphocytes [71].

Recently, we showed that myxoma virus (MYXV) has the capacity to selectively target primary human leukemia cells while spare normal HSPCs [51]. Poxviruses such as MYXV can bind and initiate entry into most mammalian cells but then discriminates permissive versus nonpermissive cells by virtue of the cell signaling circuitry of the infected cell. We have shown that upregulated AKT signaling, either as constitutive phosphorylation or induced by virus infection [72], regulates MYXV permissiveness in a wide variety of human solid tumor cell lines [73]. Considering the complexity and heterogeneity of cancer cells, this pathway is likely not the only mechanism for cancer cell specificity and there may be other mechanisms to explain the virus' discrimination between leukemia cells and normal HSPC. For example, when normal macrophages are infected with MYXV, the cells rapidly coinduce two antiviral cytokines (tumor necrosis factor and type I interferon) by a RIG-I-dependent signaling mechanism, which then aborts MYXV infection in normal somatic cells in a paracrine-like manner [74]. Thus, it could be that normal HSPCs are competent for this synergy, whereas malignant HSPCs, such as AML cells, are defective in some aspect of the tumor necrosis factor/interferon pathway. The mechanism for selective killing of cancer is still being studied, but two important factors include (1) most human cancer cells lack type I IFN and TNF synergy responses [75] and (2) most cancer cells have excessive levels of activated Akt, which facilitates MYXV replication [73]. 

## 6. Clinical Translation of Oncolytic Viruses as Purging Agents for HCT

For successful clinical translation, there are some unique requirements for oncolytic virotherapy in the setting of purging cancer cells prior to HCT. First, the OV must spare normal HSPCs. Second, the purging strategy should be simple and quick, especially when using cryopreserved stem cell products. After thawing autologous cryopreserved HSPC, cell viability decreases quickly (within minutes to an hour); thus any postthaw intervention must be quickly performed to ensure transplant of an adequate number of viable HSPC. Finally, the oncolytic virus must show limited to no infection of recipient somatic cells or tissues considering that all transplant recipients are highly immunocompromised after high-dose chemotherapy and autologous HCT.

In addition to showing preclinical safety and efficacy, the translation of oncolytic virotherapy for hematological malignancies will also require the ability to massively scale up manufacture of clinical grade virus under good manufacturing process (GMP) conditions. This process will necessitate facilities with expertise in virus production.

Currently, there are no clinical studies of oncolytic viruses as purging agents prior to autologous HCT. However, if a virus system can be optimized to meet minimum clinical criteria, then oncolytic virotherapy would have major impact in how we treat patients with blood cancers. Already, promising experimental progress indicates that early phase clinical studies of oncolytic viruses as purging agents for HCT are imminently approaching.

## Figures and Tables

**Table 1:** Oncolytic viruses for the treatment of hematological malignancies.

Virus	Disease targets	Clinical studies	References
Reovirus	MM, NHL, CLL	In development	[57]
Myxoma virus	AML, MM	In development	[51]
Measles virus	MM	Ongoing (NCT00450814)	[64]
Vaccinia virus	MM	Case report	[76]

MM: multiple myeloma; NHL: non-Hodgkin's lymphoma; CLL: chronic lymphocytic leukemia; AML: acute myeloid leukemia.
